# Left main coronary artery to right atrium fistula presenting with syncope: a case report

**DOI:** 10.1093/ehjcr/ytag516

**Published:** 2026-07-11

**Authors:** Mehak Gupta, Andrew Cole, Tom Butler, Mustafa Al-Jarshawi, Anikethana Appaji

**Affiliations:** Department of Cardiology, University Hospitals of North Midlands NHS Foundation Trust, Stoke-on-Trent ST4 6QG, UK; Department of Cardiology, University Hospitals of North Midlands NHS Foundation Trust, Stoke-on-Trent ST4 6QG, UK; Department of Cardiology, University Hospitals of North Midlands NHS Foundation Trust, Stoke-on-Trent ST4 6QG, UK; Division of Informatics, Imaging and Data Science, Faculty of Biology, Medicine and Health, Centre for Health Informatics, University of Manchester, Manchester M13 9PL, UK; Department of Cardiology, University Hospitals of North Midlands NHS Foundation Trust, Stoke-on-Trent ST4 6QG, UK

**Keywords:** Coronary cameral fistula, Transcatheter closure, Case report, Cardiac MRI, Cardiac CT

## Abstract

**Background:**

Coronary cameral fistula (CCF) is a rare coronary anomaly involving an abnormal communication between a coronary artery and a cardiac chamber or great vessel. While often asymptomatic, large fistulae may result in myocardial ischaemia, arrhythmias, or heart failure.

**Case summary:**

A 38-year-old woman presented with palpitations, dizziness, and syncope. Clinical examination revealed a continuous murmur. Transthoracic echocardiography demonstrated right-sided chamber enlargement. Cardiac magnetic resonance imaging identified a large fistula originating from the left main coronary artery draining into the right atrium, with a Qp:Qs of 1.3. Cardiac computed tomography provided detailed anatomical delineation. Coronary angiography confirmed the diagnosis and demonstrated suitability for percutaneous intervention. Successful transcatheter closure was performed using a 10 mm Amplatzer Vascular Plug II, with immediate cessation of flow and subsequent symptom resolution.

**Discussion:**

This case highlights the diagnostic value of multimodality imaging in defining fistula anatomy, quantifying shunt burden, and guiding procedural planning. Transcatheter closure is a safe and effective treatment strategy for haemodynamically significant CCF.

## Introduction

Coronary cameral fistula (CCF) is an uncommon coronary artery anomaly characterized by an abnormal communication between a coronary artery and a cardiac chamber or great vessel.^1^ The majority are congenital in origin, although acquired cases have been described following trauma, cardiac surgery, or percutaneous coronary intervention.^2^ The reported incidence in the general population is approximately 0.1%–0.2%, with over 90% of fistulae draining into right-sided cardiac chambers.^3^

## Summary figure

**Table:** 

Referred to cardiology via GP	
9/5/2024	Cardiac magnetic resonance imaging scan performed
5/6/2024	Computerized tomography of the coronary arteries performed
17/6/2024	Right and left heart catheterization performed to confirm fistula anatomy
27/11/2024	Attempt at closing of cameral fistula—failed to deliver Amplatzer vascular plug
22/1/2025	**Successful closure of cameral fistula**
4/2/2025	Re-presented to Cath Lab feeling unwell since closure—*device position assessed fluoroscopically and confirmed stable*. Found to be in Atrial Fibrillation with a rapid ventricular rate. Pharmacologically cardioverted with flecainide. Started on bisoprolol, flecainide, and apixaban
25/7/2025	7-day Holter-22 unifocal ventricular ectopics, 11 atrial ectopics, no further atrial fibrillation
11/2/2026	Repeat transthoracic echocardiogram: revealing normal left ventricular size and function. Right atrium and ventricle of normal size

The clinical presentation is heterogeneous and largely dependent on fistula size, anatomical course, and shunt magnitude. Small fistulae are frequently asymptomatic and detected incidentally, whereas larger lesions may result in chamber dilation, myocardial ischaemia due to coronary steal, arrhythmias, heart failure, or, rarely, syncope. A continuous murmur is a classical but not universal finding.

Advances in multimodality imaging, including transthoracic echocardiography, cardiac magnetic resonance imaging (MRI), and computed tomography (CT), have enhanced non-invasive characterization of fistula anatomy and physiological impact. Nevertheless, invasive coronary angiography remains the reference standard for definitive diagnosis and procedural planning.

We report a case of a symptomatic left main coronary artery–to–right atrium fistula diagnosed using multimodality imaging and successfully treated with transcatheter closure, highlighting the complementary role of cross-sectional imaging in therapeutic decision-making. This case is notable due to the uncommon anatomical origin from the left main coronary artery and the requirement for advanced loop-assisted transcatheter closure.

## Case presentation

A 38-year-old woman was referred to a tertiary cardiology clinic with a 6-month history of palpitations and intermittent dizziness, culminating in a single syncopal episode. Palpitations occurred every other day, lasting seconds to minutes, and were associated with light-headedness. The syncopal episode involved transient loss of consciousness for approximately 2–3 min without tonic–clonic activity, tongue biting, or post-ictal confusion.

She denied chest pain, dyspnoea, or reduced exercise tolerance. Past medical and family histories were unremarkable. She was a non-smoker, physically active, and took no regular medications.

On examination, a continuous machinery-type murmur was audible along the left sternal border. The electrocardiogram demonstrated normal sinus rhythm (*Figure 1*). Transthoracic echocardiography demonstrated preserved left ventricular systolic function (ejection fraction 55%–60%) and borderline right ventricular enlargement, with colour flow seen in the right atrium, labelled as a septal defect. As part of her syncope work-up, to rule out a significant arrhythmogenic cause, a 14-day ambulatory recorder was performed, which showed several runs of a likely atrial tachycardia (maximum rate 190 and duration 10 s) and 1.3% atrial ectopy.

**Figure 1:**
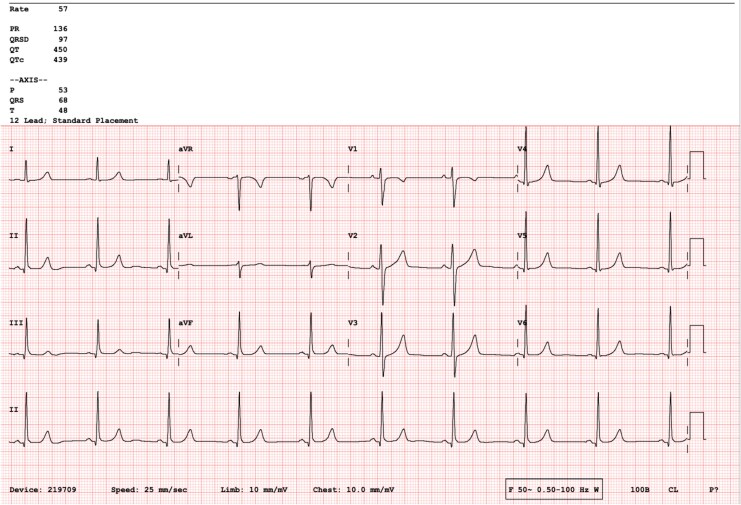
Twelve-lead electrocardiogram at initial presentation demonstrating normal sinus rhythm without significant conduction abnormality or ischaemic change.

Cardiac magnetic resonance imaging (CMR) revealed a dilated right atrium (RA four-chamber area 32 cm^2^) and ventricle (indexed 113 mL/m^2^) with preserved biventricular systolic function. The CMR identified a large fistulous communication arising from the left main coronary artery and draining into the right atrium, explaining the associated dilatation. The CMR-derived Qp:Qs ratio was 1.3 (*Figure 2*). Cardiac CT further delineated the anatomical course and spatial relationships of the fistulous tract and revealed the dimensions of the fistula to be 46 × 29 × 29 mm. (*Figure 3*).

**Figure 2:**
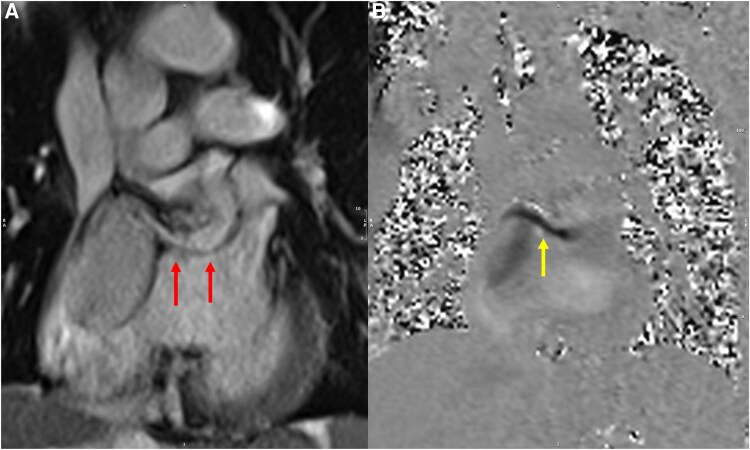
Cardiac magnetic resonance imaging demonstrating coronary cameral fistula from the left main coronary artery to the right atrium. (*A*) Still shot of steady state free precession cine image showing coronary cameral fistula extending from left main stem to right atrium (red arrows). (*B*) 2D flow image demonstrating flow across coronary cameral fistula (yellow arrow).

**Figure 3:**
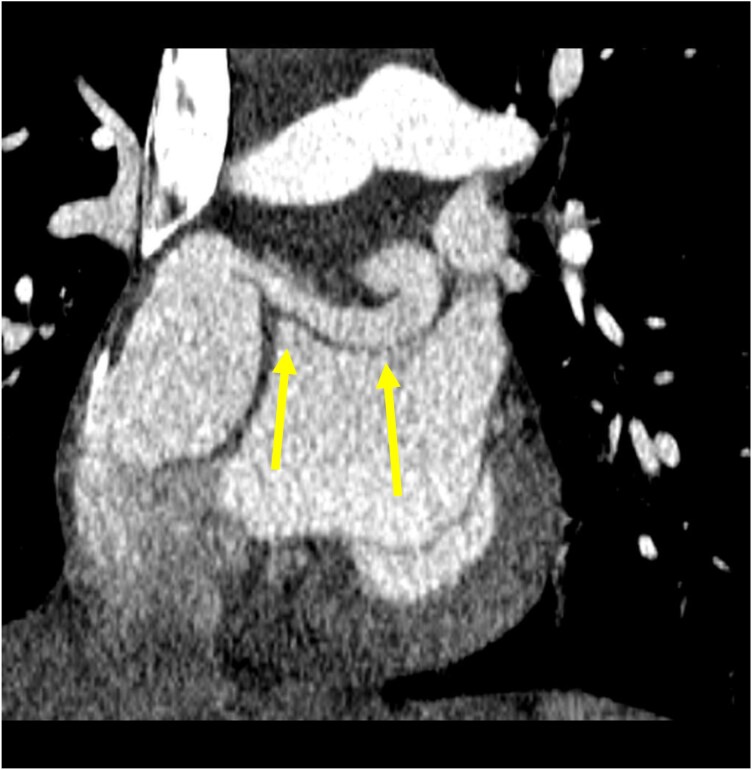
Cardiac computerized tomography delineating fistulous tract anatomy (yellow arrows).

Invasive coronary angiography confirmed a sizeable left main coronary artery–to–right atrium fistula without obstructive coronary disease. Right heart catheterization demonstrated borderline pulmonary pressures and a step-up in O_2_ saturations. (PA_mean_ 21 mmHg (normal <20, high >25 mmHg), SVC saturation 72% and PA saturation 80%).

Transcatheter closure was initially attempted via a 6-Fr right radial approach. The fistula was crossed using an Amplatzer Left 1 (AL1) guide catheter and angioplasty wire; however, instability due to 0.035-inch wire prolapse precluded stable device delivery. The procedure was converted to 7-Fr right femoral arterial access, but further attempts using AL1, AL2, and multi-purpose guide catheters were unsuccessful in achieving stable deployment of an Amplatzer Vascular Plug. Procedural parameters (contrast 210 mL, screening time 36 min, radiation dose not recorded).

A second procedure was undertaken using an arteriovenous loop technique. Access was obtained via the right radial artery and right femoral vein, and an arteriovenous loop was established within the inferior vena cava to provide enhanced support. The fistula was crossed with an AL2 guide catheter supported by a Guideliner extension catheter and angioplasty wire but delivered from the femoral vein. This approach provided improved guide catheter stability within the fistulous tract, permitting controlled device delivery. A 10-mm Amplatzer Vascular Plug II (Abbott) was successfully delivered from the femoral venous approach using an Ikari Left 3.5 guide catheter. Post-deployment angiography demonstrated immediate cessation of flow through the fistula (*Figure 4*). Procedural parameters (contrast 100 mL, screening time 9 min, radiation dose 2.9 mSv).

**Figure 4:**
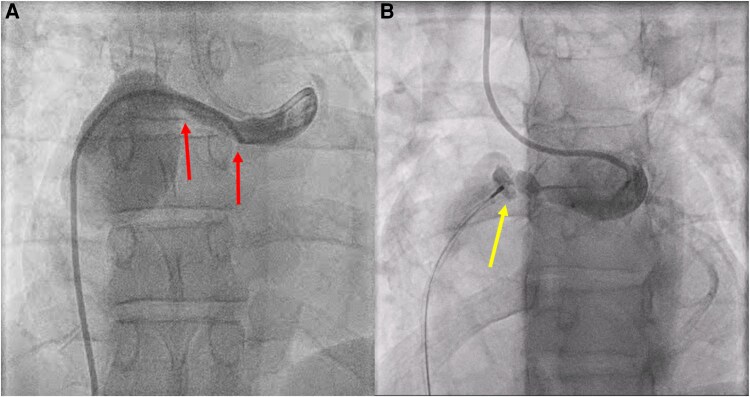
Invasive angiographic images showing flow through the coronary cameral fistula (red arrows in *Figure 4A*) with subsequent percutaneous closure with 10 mm amplatzer vascular plug II (yellow arrow in *Figure 4B*).

The patient was anticoagulated with apixaban 5 mg twice a day with a plan to continue for 3 months. Two weeks following the procedure, the patient re-presented with palpitations and fatigue. Electrocardiography demonstrated atrial fibrillation with a rapid ventricular response. Fluoroscopic assessment confirmed stable device position without residual shunt. There was no evidence of pericardial effusion or device-related complication on transthoracic echocardiography. It is possible that some of the patient’s presenting palpitations represented undiagnosed atrial fibrillation; however, transient atrial irritation related to device deployment may also have contributed. Pharmacological cardioversion with flecainide successfully restored sinus rhythm. She was commenced on bisoprolol 2.5 mg once a day and flecainide 50 mg twice a day for rhythm control, alongside continued anticoagulation with apixaban.

A 7-day ambulatory Holter monitor at 6 months demonstrated sinus rhythm throughout, with infrequent unifocal ventricular ectopics and occasional atrial ectopics, but no recurrence of atrial fibrillation. Repeat transthoracic echocardiography at 1 year showed normal left and right ventricular size and function, with no residual shunt and normal right atrial dimensions. At the most recent clinical follow-up, she remained asymptomatic with complete resolution of palpitations and no further syncopal episodes.

## Discussion

CCFs are a rare entity, reported in approximately 0.1%–0.2% of the population.^3^ More than 90% drain into right-sided cardiac chambers.^4^ Small fistulae are often incidental findings and remain clinically silent; however, larger or haemodynamically significant lesions may produce a broad spectrum of symptoms, including exertional dyspnoea, fatigue, chest discomfort, arrhythmias, or overt heart failure.^5^ Myocardial ischaemia may occur due to the ‘coronary steal’ phenomenon, whereby blood is preferentially shunted through the low-resistance fistulous pathway, reducing distal coronary perfusion and potentially predisposing to arrhythmogenesis.^6^ The classical murmur associated with CCF is described as a loud, continuous murmur that peaks in mid-to-late diastole, best heard at the mid-to-lower sternal border, but can be dependent on the site of fistula drainage.^1^

Transthoracic echocardiography serves as an appropriate first-line investigation and may demonstrate chamber enlargement or abnormal flow patterns. Transoesophageal echocardiography offers improved sensitivity in adults.^6^ Cross-sectional imaging with cardiac CT or MRI provides detailed anatomical delineation, spatial orientation, and assessment of shunt burden. In the present case, cardiac MRI facilitated initial detection and physiological assessment. Nevertheless, invasive coronary angiography remains the reference standard for definitive anatomical confirmation and procedural planning, allowing evaluation of fistula origin, course, haemodynamic impact, and suitability for transcatheter closure.

Management strategies are guided by symptom burden and haemodynamic significance. Asymptomatic, small fistulae may be managed conservatively with serial imaging surveillance to monitor for progressive dilatation of the feeding vessel or chamber enlargement.^7^ In contrast, large or symptomatic fistulae warrant closure to prevent long-term complications. Transcatheter occlusion has become the preferred approach in suitable anatomical settings, while surgical repair is generally reserved for complex anatomy or selected paediatric cases in whom percutaneous access is not feasible.^8^

There is relatively sparse literature regarding arrhythmias following coronary cameral fistula closure given the rarity of the condition. Reported arrhythmias include atrial fibrillation and ventricular arrhythmias.^9^ Atrial fibrillation appears uncommon but may occur in patients with pre-existing right atrial dilatation or atrial stretch.^10,11^ In the present case, early post-procedural atrial fibrillation may have reflected transient atrial irritation related to device deployment or early reverse atrial remodelling following abrupt haemodynamic correction. The absence of documented atrial fibrillation prior to closure, lack of recurrence on extended ambulatory monitoring, and subsequent normalization of right-sided chamber dimensions support a transient rather than persistent arrhythmogenic mechanism.

## Conclusion

This case illustrates the pivotal role of multimodality imaging in diagnosing and planning treatment of coronary cameral fistulae. Transcatheter closure is a safe and effective therapy for haemodynamically significant lesions, providing symptomatic relief and preventing long-term sequelae.

## Data Availability

No new data were generated or analysed in support of this research

## Lead author biography

Dr Gupta is a Cardiology Consultant working in the West Midlands and has a subspecialty interest in Cardiac Imaging

## Notes

**Consent:** The authors confirm that written consent for submission and publication of this case report including images and associated text has been obtained from the patient, in line with COPE guidance
